# The Utility of Our Journal

**Published:** 1871-11

**Authors:** 


### THE UTILITY OF OUR JOURNAL.

Perhaps the shortest, most expressive, and most comprehensively truthful notice ever yet made of our pet, is from the pen of the editor of "The Canadian Independent," for September, 1871. "Hall's Journal of Health is perpetually under its contemporaries' scissors." A recent number of the "Galaxy," in an objurgation, refers to it as "that much-quoted periodical." That at least is the idea, if not the words. On several occasions we have asked long-time friends if they took the Journal, wishing to make some reference to it; and have received the very uniform reply, "No, for all you write seems to be quoted in our other papers, so we get the reading of it for nothing." We have recently, as before, seen three or four or more extracts from it in a single newspaper. Articles are constantly copied all over the country without credit; but this is unavoidable in the hurry of making up a newspaper, especially the dailies; the only inconvenience to us is, that we are attracted by the headings, read a line or two, go on, thinking it is strikingly true, and by the time we get to the bottom, the idea is completed, that we wrote it ourselves. And we state it as something singular in the working of the mind, that a writer should read five, ten, twenty lines from his own pen, before the mind takes hold of it, certain that it is its own child.

When an article is copied without credit, some other paper copies that, and gives the other or its editor, credit as the author. In one case we read an article credited to "Dr. Spicer;" that was six or more years ago; and it is going the rounds yet; but we have never yet been able to decide whether we wrote it or not. Some of the sentences are verbatim, but now and then comes a word which we do not remember ever to have used.

Another curious thing: Every man has his own set of words, and seldom goes out of that set for a choice. But perhaps every one who writes much will now and then feel the want of some word outside of his own set, which would express his idea better than any of his own, and he uses it. We have noticed in our own mental operations that even now a word comes to us, which we know on the instant that we have never used before.

The choice of words is different in different minds, temperaments, and moralities. The vulgar and low-bred use slang phrases; the cultivated employ refined, delicate expressions. The systematic select words which are definite in their meaning. The young, the unchastened, those who have no definite views, who are of excitable temperaments, deal largely in exaggerations; everything is "awful," "splendid," "magnificent." When a writer sees an article expressing his own ideas, especially if the words are pretty much of his own set, he warms up to the author at once, and feels intense satisfaction, the next in degree to that which he evinces in reading what he has written himself, when he sees it in print. The daily and weekly hacks have not time for these enjoyments.

Some writers cannot bear to read their manuscript after it has been written, not even for review; this is very often the case. Sometimes the interest comes only when they have seen it quoted; with this proof of appreciation, it is read with pleasure. At other times, a writer becomes hypercritical of his own composition; and reads it over and over again; adding a word here, erasing another there. At one place interlining to throw in another idea; at another, blotting out words, lines, and sentences as wholly superfluous. Now and then, after all this trouble, he becomes disgusted with the article, tears it up into a thousand pieces, then writes the whole thing anew, if he must write on it; if not compulsory, he takes his hat, clears out, and don't resume his pen for a week, unless he is obliged to; if so, he selects some wholly different subject.

Sometimes an article, or a book even, will be written for publication, but nobody will touch it with a ten-foot pole; pay for its printing, or publish the book yourself, and the same rejected article will have the run of the whole periodical press; or if a book, it will sell by thousands. Such has been our own experience, and doubtless that of other writers. It has afforded us real fun many times, to have publishers write to us for an article. We send it, forget all about it; hear in a short time that it is "not up to the mark," or something of that sort. We put it in the JOURNAL, and away it goes, from Maine to Florida; from Cape Cod to the Golden Gate, and sometimes keeps on going, and like the man with a cork leg, it won't stop. We wrote one book. Printers didn't think it would take. But we knew it was a want of the hour, with nothing to meet that want. Forty thousand copies of it were sold.

These things are written, not that they pertain to health, in fact we began this article expecting it to be disposed of in a single paragraph, but one thing led to another. Nevertheless, writers will feel deeply interested in the statements, and many may find encouragement in some of the suggestions made, at least in one direction — the rejection of an article, even by a Solomon, is no evidence that it does not possess merit.

